# Current Concepts and Unresolved Questions in Dietary Protein Requirements and Supplements in Adults

**DOI:** 10.3389/fnut.2017.00013

**Published:** 2017-05-08

**Authors:** Stuart M. Phillips

**Affiliations:** ^1^McMaster University, Hamilton, ON, Canada

**Keywords:** sarcopenia, critical illness, chronic illness, lean body mass, leucine, creatine

## Abstract

Protein needs for otherwise healthy individuals older than 19 years are defined by the recommended dietary allowance (RDA) at 0.80 g protein/kg/day. There is no recommendation in the current RDA for subpopulations of older adults or people in various pathological situations. Despite the lack of a separate recommendation, there exists a growing body of evidence that is strongly suggestive of an increased need and/or benefit for protein in older persons. That is, intakes beyond the RDA are, in older persons, associated with benefits. In addition, a number of catabolic states including critical illness also result in a sharp elevation in the needs for protein and amino acids. An underappreciated issue in protein nutrition is the impact of protein quality on clinically relevant outcomes. The introduction of a new protein scoring system—the digestible indispensable amino acid score (DIAAS)—for protein quality has raised a forgotten awareness of protein quality. The DIAAS, which replaces the protein digestibility-corrected amino acid score (PDCAAS), is based on ileal digestibility of protein and a different test protein than PDCAAS and has values greater than 1.0. The aim of this article is a brief review and summary recommendations for protein nutrition and protein requirements in populations who would benefit from more protein than the RDA. The emphasis of the review is on muscle protein turnover, and there is a discussion of the impact of protein quality, particularly as it applies to commercially available protein sources. The evidence for more optimal protein intakes is considered in light of the potential health risks of consumption of protein at levels greater than the RDA.

## Introduction

Body proteins are constantly being turned over. This constant synthesis and degradation of proteins provides for a mechanism of protein maintenance in the event that proteins are damaged due to oxidative stress, protein misfolding, or other processes [for review see Ref. (1)]. The flux through protein synthesis and degradation is affected by a number of variables including age, activity level, sex, hormones, disease, and diet (2). However, gains or losses in body protein mass, mostly measured as a reduction in lean body mass (LBM), are due to acute and chronic imbalances in protein turnover (3). Thus, the relative rates of protein synthesis and breakdown and their effectors are important to understand in the context of any change in LBM. What is also important to realize is that the relative rates of protein turnover in various tissues differ markedly. For example, muscle protein turnover proceeds at a rate of 0.06%/h (~1.5%/day) (3), blood protein turnover rates vary markedly from 1 to 30%/h (4), and intestinal protein turnover rates vary from 1 to 2%/h (5). Thus, contribution to total body protein turnover, which would be a function of the protein turnover rate and the size of the protein pool, would vary substantially. The ingestion of protein and subsequent hyperaminoacidemia result in a stimulation of protein synthesis, and this appears to be true regardless of the anatomical and subcellular protein pool (6) being studied (7). Feeding-induced hyperaminoacidemia and the transient stimulation of protein synthesis result in a transient 3- to 4-h period of net protein accretion (3). During fasted periods, the relative hypoaminoacidemia results in a reduced stimulation of protein synthesis and protein breakdown is, comparatively, elevated (3). Thus, acute feeding and fasting result in transient (hours) fluctuations in protein synthesis and breakdown of proteins (3). There are also chronic (weeks, years) periods of protein gain during growth, pregnancy, and muscular hypertrophy. In contrast, there are chronic periods of protein loss that occur during disease, with aging, and during inactivity (bed rest, immobilization) (3).

Protein requirements for healthy persons older than 19 years are set according to the recommended dietary allowance (RDA), which currently stands (and has remained unchanged for a number of years) at 0.80 g/kg/day (8). A number of reports have challenged the concept of the RDA as a minimal requirement rather than a “recommended” intake of protein that is “allowed” and questioned whether more optimal health outcomes could be achieved with protein intakes greater than the RDA. Implicitly, the acceptable macronutrient distribution range (AMDR), stating that anywhere from 10 to 35% of total energy intake can come from protein, is a tacit endorsement that protein intakes at levels higher (much higher) than the RDA are associated with good health. While nuanced in details the general assessment of why dietary protein intakes greater than the RDA are optimal (as opposed to minimal) are based around assessments of appetite regulation, weight loss and maintenance, and LBM preservation [for reviews, see Ref. (9–11)]. Balanced against a recommendation for protein intakes that are greater than the minimal level are health concerns around renal and bone health.

The aim of this short review is to examine the basis of protein requirements and to review evidence that points to benefits of protein intakes greater than the current RDA. The focus is on healthy adults, the elderly, and critical illness. In the context of understanding protein requirements, a brief discussion of protein quality under the older [protein digestibility-corrected amino acid score (PDCAAS)] and newer [digestible indispensable amino acid score (DIAAS)] scoring systems is also presented.

## Protein Requirements

Current protein requirements set the RDA at 0.80 g/kg/day, and this is based on nitrogen balance (8). Nitrogen balance has been used for more than 60 years to establish protein requirements, and the balance methodology is used not only for protein but also for a number of other nutrients. There have long been recognition of the shortcomings of nitrogen balance (12) yet it is the methodology that underpins the RDA, a value that is used by many in nutritional practice as a target for protein intake. While the discussion may be semantic, the term RDA would inherently imply that the protein intake is “recommended,” and it is all that you are “allowed” to eat. Of course, neither is strictly speaking true, and the definition of the RDA is that it “…is an estimate of the minimum daily average dietary intake level that meets the nutrient requirements of nearly all (97 to 98 percent) healthy individuals in a particular life stage and gender group” (p. 24). A pertinent question is whether there is benefit to consuming protein at a level that is above the minimum? The RDA for protein has been challenged as a concept that should drive protein intakes (13). It was pointed out by Wolfe and Miller that the AMDR for protein at 10–35% of energy coming from protein is concept that is more in line with an optimal rather than a minimal dietary strategy. Nonetheless, a comparison of the protein requirement (RDA) of a 55-year-old man who is 1.80 m, weighing 80 kg, at 64 g protein/day versus the range of protein intakes from the AMDR of 65–228 g protein/day (assuming an energy requirement of 2,600 kcal/day or 10.9 MJ/day) reveals quite a disparate recommendation. The Institute of Medicine states that the AMDR “…is provided to give guidance in dietary planning by taking into account the trends related to decreased risk of disease identified in epidemiological and clinical studies” (p. 29). Thus, there are, according to the AMDR, protein intakes that are well above the RDA (up to 2.8 g/kg/day) that are associated with good health. Interestingly, an alternative interpretation, applying a bi-phase linear regression as opposed to a linear regression model, of the same data used to establish the RDA for protein (14) has come to the conclusion that even minimal protein requirements for healthy young men (and presumably women) should be 1.0 g/kg/day (15) and as high as 1.2 g/kg/day (16). Thus, there is disagreement over whether 0.8 g/kg/day is a minimum value and should be the RDA (14) or whether protein intakes of 1–1.2 g/kg/day (15, 16), using nitrogen balance, are a minimum intake. As a variable, age was considered in the original formulation of the RDA for protein, and Rand et al. (14) concluded that, “…there was a difference of almost 27 mg N/kg/d in the median requirement; however, the difference was not statistically significant.” Relevant to a discussion of protein requirements in the elderly is the fact that the data used to formulate this conclusion (14) came from only 14 older (older than 67 years) men and women (seven of each).

The indicator amino acid oxidation (IAAO) method for determining protein requirements has been developed as an alternative to nitrogen balance. While an extensive discussion of the IAAO method, and its assumptions, is not warranted here, the interested reader may wish to refer to several reviews on the topic (17, 18). The IAAO method has consistently yielded higher estimates for protein requirements than nitrogen balance (15, 19), particularly in the elderly (20–22). From these studies, a safe minimal protein intake would be 1.0–1.2 g/kg/day for normal healthy younger persons (16) and at least 1.2 g/kg/day and perhaps as high as 1.4 g/kg/day for older persons (20–22). Of course, even these estimates are minimal not optimal, and these intakes need to be tested in longer term trials to establish the longer term ramifications of consuming a protein intake at this level. Of relevance to longer term health outcomes for older persons is the impact of consuming protein at levels of at least 1.2 g/kg/day on LBM. This point is emphasized to even greater degree when one considers that 25% of older men, and up to 50% of older women, are not consuming the protein RDA let alone a protein intake of 1.2 g/kg/day (23).

## Protein Quality

The RDA for protein at 0.80 g/kg/day is recommended to come from mixed sources of proteins of generally high quality. Until recently, protein quality was estimated using the PDCAAS. An expert recommendation has, however, been that the PDCAAS be replaced in favor of a new scoring system called the DIAAS (24). There are some notable differences between PDCAAS and DIAAS, and the main reasons to advocate DIAAS as a replacement for PDCAAS are around the shortcomings of the PDCAAS method, specifically:

1. protein digestibility-corrected amino acid score uses fecal protein digestibility, and there is significant bacterial (colonic) metabolism of amino acids that can falsely enhance values of true protein digestibility;
2. truncation of PDCAAS values at 1.0 does not account for the bioavailability of individual indispensable amino acids that may have specific roles, and thus, proteins of higher quality are not identified;
3. protein digestibility-corrected amino acid score values are overestimated because of limited bioavailability of specific forms of amino acids such as lysine (25); and
4. fecal protein digestibility values are determined using rats, which have a different requirement for amino acids for growth and maintenance versus humans.

Table 1 lists the PDCAAS and DIAAS of some commercially available isolated protein sources and some commonly consumed protein-containing foods. The limiting amino acids in the proteins and foods listed in Table 1 differ, but an important point is the reference for PDCAAS is egg protein, whereas in DIAAS, it is a theoretical best protein. The implementation of DIAAS will, however, take time, and a number of proteins and protein sources will have to be examined for their ileal digestibility. Ileal digestibility would be very difficult to measure in humans and even so in animal systems (24), and it may be more practical to rely on *in vitro* digestion methods to establish digestibility.

**Table 1:** **PDCAAS and DIAAS scores for selected isolated proteins and foods**.

Food	PDCAAS	DIAAS	Limiting AA
MPC^a^	1.00	1.18	Met + Cys
WPI^a^	1.00	1.09	Val
SPI^a^	0.98	0.90	Met + Cys
PPC^a^	0.89	0.82	Met + Cys
RPC^a^	0.42	0.37	Lys
Whole milk^b^	1.00	1.43	Met + Cys
Cooked peas^a^	0.58	0.82	Met + Cys
Cooked rice^a^	0.62	0.59	Lys
Almonds^e^	0.35	0.40	Lys
Chickpeas^f^	0.52	0.67	Trp
Tofu^c^^,^^d^	0.70	0.97	Met + Cys
Corn-based cereal^a^	0.08	0.01	Lys
Hydrolyzed collagen^g^	0.0	0.0	Trp

*PDCAAS, protein digestibility-corrected amino acid score; DIAAS, digestible indispensable amino acid score for adolescents and adults; MPC, milk protein concentrate; WPI, whey protein isolate; SPI, soy protein isolate; PPC, pea protein concentrate; RPC, rice protein concentrate*.

*^a^Values from Ref. (26)*.

*^b^Values from Refs. (24, 117)*.

*^c^Values for PDCAAS from Ref. (118)*.

*^d^Values for DIAAS from Ref. (119)*.

*^e^Values from Ref. (120)*.

*^f^Values from Ref. (121)*.

*^g^Hydrolyzed collagen has a PDCAAS and DIAAS score of 0 since it contains no tryptophan (Trp) and is very low in methionine (27)*.

Hydrolyzed collagen has become a mainstay in a number of supplements, and it has a quality score of 0 unless supplemented with tryptophan. Even with tryptophan supplementation, collagen has a very low quality score (Table 1). An often cited benefit of collagen supplementation is that since it is derived from bone and cartilage, it contains all of the amino acids necessary to support the health of these tissues. However, it is important to realize that most amino acids contained in hydrolyzed collagen are non-essential [i.e., glycine (Gly), proline (Pro), alanine (Ala)] or posttranslationally modified [i.e., hydroxyproline (HyPro)], and thus, there is not a strong case to be made for supplementation with a protein source that contains amino acids that adults can readily synthesize (Gly, Pro, Ala) or that cannot be utilized due to the lack of a tRNA for the amino acids, which is the case for HyPro. Nonetheless, collagen appears in a number of supplements targeted at older persons (28), which is in opposition to the recommendation that older persons consumed a higher protein and leucine (see below) intake. As Castellanos et al. stated, “…collagen-based supplements that provide insufficient amounts of many IAAs when compared with the 2005 DRI reference pattern are not considered to be ‘complete’ proteins” (28).

## Leucine

An important consideration relevant to protein quality is the recognition that leucine, the indispensable amino acid, is not only a building block for protein synthesis but also, in muscle, a trigger for protein synthesis (29–32). The existence of an intracellular leucine “trigger” (3, 33–35) predicts that postprandial leucinemia and the ensuing increase in intracellular leucine concentration is stimulus that results in a rise in muscle protein synthesis (MPS). This stimulation appears to be *via* the leucine-binding protein Sestrin2 (36, 37), which results in activation of the mechanistic target of rapamycin-complex 1 (38). Therefore, stimulation of MPS would require ingestion of a protein that is higher in leucine or fortification of a lower leucine-containing protein (i.e., lower quality or lower dose) with leucine (34, 35). In fact, part of the FAO recommendations on protein quality were that individual amino acids be treated as nutrients (24), and in the case of preservation or restoration of lost muscle mass, leucine would be an important or perhaps the most important amino acid in stimulating MPS (3, 33–35). The amino acid content of proteins using the DIAAS-based ideal protein yield individual amino acid reference ratios (AARRs), which are ratios of each indispensable amino acid in a protein relative to the ideal protein. Given the importance of leucine for stimulating MPS and driven muscle protein retention, the AARR for leucine is shown for seven commonly consumed supplemental proteins (Table 2). Importantly, in none of the proteins highlighted in table is leucine considered the rate limiting amino acid (Table 1) in normal healthy persons; however, it is worth appreciating that in states of muscle loss (aging, critical illness), leucine would become of paramount importance. Thus, considerations of protein quality by DIAAS aside, the leucine content of a protein would also be important to consider, and this appears to be particularly true in aging (39). Thus, as Table 2 highlights milk-based proteins would be, gram-for-gram, better sources of protein to provide to support the delivery of leucine to support MPS. Such a recommendation would be particularly relevant in situations where protein intake is restricted (for example, in kidney disease) and limited (such as in older persons with a limited appetite) and in situations of pronounced catabolism, which is commonly observed in patients in intensive care.

**Table 2:** **Leucine amino acid reference ratio (AARR) for selected isolated proteins**.

Protein	Leucine AARR
Milk protein concentrate	1.77
Whey protein isolate	2.57
Whey protein concentrate	1.93
Soy protein concentrate	1.29
Pea protein concentrate	1.37
Rice protein concentrate	1.11

*Values from Ref. (26)*.

The elderly appear to have a greater requirement for leucine to stimulate protein synthesis than their younger counterparts meaning that older persons would need to be provided with greater intakes of protein or leucine to stimulate MPS and, presumably, retain muscle (39–42). Hence, proteins that contain a high percentage of leucine would be advantageous to stimulate MPS and promote lean mass retention (43). An important consideration is that higher protein/leucine to activate MPS and stimulate retention of muscle could be thus achieved with lower protein intakes if higher leucine-containing protein were consumed. This may be important for the elderly in whom energy intake is relevant, and lower protein intakes may avoid a potential appetite suppression (44). A remaining research question is whether diets higher in leucine could be used in a chronic setting to attenuate the loss of muscle mass and function. Results from recent sufficiently long and an appropriately powered trial indicate that this may be the case (43). A twice daily dietary supplement for 13 weeks containing whey protein, leucine, and vitamin D (20 g whey protein, 3 g total leucine, and 800 IU vitamin D) was given to older (~78 years) primarily independent-living sarcopenic adults and resulted in improved chair–stand test time and showed a greater gain in appendicular muscle mass than the control group (43). It is not possible to isolate the results of this trial to protein/leucine alone due to the added vitamin D (43); however, the impact of vitamin D on muscle mass and function appears to be minimal (45) or is restricted to older persons with deficient levels of vitamin D (i.e., less than 30 nmol/L) (46). In addition, a separate trial using a similarly formulated protein/leucine vitamin D-enriched supplement showed similar benefits (47). A meta-analysis examining the impact of leucine-based supplements concluded that sarcopenic patients taking leucine-based supplements gained LBM, but did not show gains in either grip or leg strength (48).

## Aging

Aging is associated with a slow loss of muscle mass and function, termed sarcopenia. Sarcopenic muscle loss proceeds at a rate of ~0.8%/year, and strength is lost at a rate of ~1–3%/year (49, 50). It is difficult to say exactly when sarcopenia begins, but it likely that in the fifth decade of life that muscle mass and function begin to measurably decline dependent to large extent on a persons’ level of physical activity and general health (49, 50). Nonetheless, as potent drivers of muscle protein turnover, physical activity and dietary protein can be manipulated to attenuate losses in LBM. Clinical observations of changes in LBM give credence to a protein-/leucine-driven thesis of muscle retention and support the recommendation for greater protein/leucine in older persons to stimulate MPS and aid in retention of LBM (51, 52). For example, men and women (*n* = 2,066) aged 70–79 years in the highest quintile of protein intake (~1.1 g/kg/day, 18.7% energy from protein) lost approximately 40% less LBM and appendicular (arms and legs) LBM than did those in the lowest quintile of protein intake (~0.7 g/kg/day, 11.2% energy from protein) (53). While the associations were attenuated slightly after adjustment for change in fat mass, the results remained significant (53). McDonald et al. (54) reported that dietary leucine intake was associated with LBM change in older (>65 years) persons across 6 years of follow-up. Older participants in the highest quartile of leucine intake (7⋅1 g/day) experienced LBM maintenance, whereas lower intakes were associated with LBM loss over 6 years. This relationship was not modified by sex or the presence of cardiovascular disease. The authors concluded (54) that a greater leucine intake in conjunction with adequate total protein intake was associated with long-term LBM retention in a healthy older Danish population. These observations (53, 54) are similar to those reported in other studies (55, 56); primarily, higher protein intakes result in a greater net retention of LBM in older persons.

An important point, however, is that preservation of muscle mass while laudable is important only if it is associated with an improvement in strength and function. An effect of protein supplementation on muscle mass and function has been seen in some protein/leucine intervention trials (43, 47) and observational data (57). In addition, cross-sectional analysis of dietary intake data has shown a relationship between total (57) and per-meal protein intake on not only muscle mass but also muscle function (58). In fact, the concept of not just daily protein intake but per-meal protein intake, to provide a more optimal per-meal stimulation of MPS, may be an important area of research especially in the elderly (59). A recent trial in middle-aged adults showed that supplementation of the breakfast and lunchtime meals, meals lower in protein in many societies, was able to promote LBM accretion (60).

## Critical Illness

Critical illness is characterized by a pronounced rapid reduction in LBM (61, 62). This reduction is due to a combination of inactivity, hypercytokinemia, and hypercortisolemia that reduce MPS and increase muscle protein breakdown (MPB) (62, 63). A number of clinical guidelines recommend protein intakes in critical illness (64, 65) of at least 1.2–1.5 g/kg/day. These recommendations have been challenged, however, as being too low, and Hoffer and Bistrian have recommended protein provision of 2–2.5 g/kg/day for critically ill patients (66, 67). This level of protein intake is rationalized by these authors as being necessary to offset the sharp negative nitrogen balances seen in critically ill patients (66, 67). It has been argued by the same authors that it is not appropriate to simply feed the critically ill more energy (68) and that protein is the most important macronutrient (66, 67) to provide substrate for all protein-requiring processes, which would be under the influence of pro-catabolic and anti-anabolic stimulation. Nonetheless, an analysis of nitrogen balance in older intensive care unit (ICU) patients showed that they were refractory to lower protein intakes and that it was not until protein intakes that were greater than 2 g/kg/day that nitrogen balance became positive (69, 70). The lack of response of whole-body nitrogen balance in older ICU patients (69, 70) is similar to what has been seen in healthy older persons with respect to MPS (71). Namely, in healthy older persons, there is a lowered anabolic response to lower protein intakes, but at higher protein intakes, there is a stimulation of protein synthesis (71) and/or a suppression of proteolysis (69, 70), which results in improved protein balance. The case for higher protein intakes in the critically ill (66, 67) currently lacks clinical trial support, but appears to have some support in the elderly (69, 70).

The potential for ureagenesis and azotemia leading to impairments in renal function is a concern on patients in the ICU, particularly the elderly. According to Dickerson (70), the survival advantage, at least based on the observational data, in those receiving higher protein intakes (72–74) would be a strong indication that renal function is less important. Instead, Dickerson says, “…the limitation of protein intake on a short-term basis is unwarranted in the patient without overt acute kidney failure… The whole theoretical point of compromising renal function with higher protein intakes is moot if the patient is not given a sufficient protein intake in an effort to survive the acute insult that led to ICU admission” (70). It would of course be beneficial to have better clinical data on higher versus lower protein intakes in critically ill patients and to show improved survival balanced against measures of renal function.

## Pressure Ulcers and Acute and Chronic Illness

Two areas in which protein provision has been examined and postulated to play a beneficial role are in pressure ulcer healing and acute illness. Pressure ulcers occur in up to 10% of hospitalized patients and are potentially fatal particularly in older more frail patients (75). Expert consensus recommendations for pressure ulcer healing are for a protein provision of 1.25–1.5 g/kg/day, which received a positive recommendation based on clinical trial evidence (76). While recommendations for pressure ulcer healing are for nutrition to be delivered *via* normal dietary sources, this is not always feasible. Several trials using arginine-enriched protein-containing supplements have been shown to yield positive results on pressure ulcer healing (77–79). In addition, a trial of 1.2 versus 1.6 g/kg/day of protein resulted in improved pressure healing (80). The most recent meta-analysis of data for nutritional support of pressure ulcer healing included 23 randomized control trials (RCTs) and examined a variety of supplements (81). While the supplements were mixed in terms of composition, there were 11 of the 23 RCTs in which a mixed nutrient supplement containing protein alone and mixed supplements of protein, vitamins, carbohydrate, and lipids were studied. All 11 studies compared the nutritional intervention with a standard intervention, for example, standard hospital diet, or standard hospital diet plus placebo. The authors stated, “The clearest conclusion that can be drawn… is that it remains unclear whether nutritional supplementation reduces the risk of pressure ulcer development” (81). Similar conclusions were reached for pressure ulcer healing (81). Thus, while protein needs appear to be elevated in patients undergoing wound healing (13) and recommendations are for higher protein intakes in pressure ulcer healing (76) and acute illness in older persons in general (51), the use of high-energy protein-containing supplements is equivocal at this time (81). Clearly, larger trials with attempts to assess both ulcer incidence and ulcer healing and targeted protein intakes would be valuable.

The benefits of protein supplementation with acute illness are not readily apparent. Only a few small trials have attempted to improve protein intake in people with acute illness (51, 82, 83). In general, protein-containing supplements result in small improvements in outcomes that are more marked when combined with programs of rehabilitation/physical activity (82). In malnourished or undernourished patients, some outcomes are improved, and meta-analyses have shown (84, 85) improved nutritional status. The interventions used in the trials (84, 85) aimed to provide between 175 additional kcal/day and up to a maximum of 1,350 additional kcal/day. Additional protein was between 10 and 50 g protein/day (84, 85). Some outcomes were improved with protein/energy supplementation (85). Most notably, older undernourished or malnourished persons showed, with protein/energy supplementation, weight gain, reduced mortality, and a reduced risk of morbidity [infectious complications, total pressure sores, patients too ill to continue, exacerbation of chronic obstructive pulmonary disease (COPD), anesthetic, surgical infection, hospital readmission, incomplete wound healing, prescription of antibiotics, and total severe adverse events] (85). There was no detectable effect of protein/energy supplementation on functional outcomes (85), which likely requires either the addition of a structured activity program (51) or at least needs to be adequately powered to detect such outcomes (43).

Meta-analyses of nutritional supplementation in patients with stable COPD have reported a benefit (86). The main outcomes improved in stable COPD patients with protein/energy supplementation were increases in LBM and weight gain especially if malnourished. There was also low-quality evidence that patients with COPD were able to improve their exercise capacity (6-min walk test) and also their health-related quality of life (86). Since low body weight and muscle mass are common in people with COPD as well as some degree of malnutrition, the improvements seen are encouraging (86). Interestingly, pulmonary and physical rehabilitation has been shown to relieve dyspnea and fatigue and enhanced the sense of control that individuals have over their condition as well as improving their health-related quality of life and exercise capacity (87). Given the potential synergy between exercise and protein supplementation, it would be interesting to determine whether a synergism between protein and pulmonary rehabilitation exists.

Currently, consensus guidelines recommend a higher protein intake for patients with pressure ulcers (76). Existing evidence does not support a role of multiingredient supplements providing protein on pressure ulcer incidence or healing (81).

What appears clear is that protein–energy-containing supplements are more likely to have measurable impacts in older undernourished or malnourished individuals and in those who are sarcopenic. The impact of these supplements on functional outcomes is small, if present at all, and would likely require the addition of an activity/exercise program, which may be synergistic with protein supplementation in enhancing outcomes (88).

Patients with stable COPD derive small but clinically meaningful benefits from protein/energy supplementation. It is unclear in most trials whether outcomes are due to energy provision *per se* or protein provision, although as protein is a functional substrate as well as potentially being active in signaling and metabolism, it is likely that it is protein *per se* and not total energy intake that is responsible for observed changes.

## Adjunctive Ingredients

There are a number of adjunctive ingredients that have been added to protein formulations to aid in outcomes related to skeletal muscle retention and function. The discussion here is focused on two ingredients for which there is enough evidence to discuss relevant outcomes: beta-hydroxy-beta-methylbutyrate (β-HMB) and creatine. Humans have a limited capacity to endogenously synthesize β-HMB, a metabolite of the amino acid leucine (89, 90), that has been extensively studied in clinical trials and shown to have small impacts on gains in LBM (89–91). Leucine and β-HMB have parallel modes of action in terms of stimulating MPS (92) and inhibiting MPB (93) although the signaling pathways may be distinct (92). Thus, it would be expected that by comparison to protein that provides leucine, the impact of β-HMB would be minimal; however, in clinical trials, β-HMB has most often been compared to a mixture of dispensable amino acids (DAAs) or a non-caloric placebo (89, 90). In an attempt to meta-analyze the impact of β-HMB on the elderly, Molfino et al. (90) concluded, “Meta-analysis was not feasible due to the considerable variation in study design, the type and timing of HMB intervention…” and importantly in the same review, the authors stated, “Quite surprisingly, studies evaluating the effects of HMB alone in old adults were limited.” Thus, the effects of β-HMB in older adults are limited in terms of what can be concluded as to effect of the supplements on LBM and muscle function, a conclusion supported by Fitschen et al. (91). Despite the conclusion reached by Molfino et al. (90), Wu et al. (89) did perform a meta-analysis of β-HMB and its effects in the elderly. These authors included 7 RCTs with 147 older adults receiving β-HMB and 140 receiving a non-caloric or DAA control placebo (89). The meta-analysis showed greater muscle mass gain in the β-HMB (standardized mean difference = 352 g; 95% confidence interval: 110–594 g). What is hard to reconcile is how the studies included in this analysis (89) could truly constitute a valid meta-analysis given the heterogeneity of the included interventions (including other potentially active amino acids including arginine and glutamine in older subjects undergoing bed rest, women only, subjects undertaking resistance exercise training, and in patients with solid tumors). Despite a trivial effect of β-HMB on muscle mass, there were no improvements in muscle function (89).

Deutz et al. (94) supplemented a variety of older malnourished patients who were hospitalized (congestive heart failure, acute myocardial infarction, pneumonia, or COPD) with a protein-containing supplement that contained β-HMB. Compared to the placebo group, the protein supplemented group showed no difference in 90-day readmission rate, but 90-day mortality was significantly lower. Protein supplementation resulted in improved odds of better nutritional status at 90 days and an increase in body weight at 30 days. There was no effect of supplementation on length of hospital stay or on function as assessed by activities of daily living (94). Due to the lack of a control group that did not consume β-HMB, but did consume the protein and energy, it was not possible to isolate the effect of the treatment to β-HMB. It appears, based on data from protein/energy supplements (85), that the effect of the protein energy supplement was due to increased energy and/or protein provided to the supplemented versus minor constituents such as β-HMB and/or vitamin D (95).

Creatine has been shown in meta-analyses to enhance exercise-induced gains in LBM as well as strength and function (96). It has also been postulated that creatine may be a functional ingredient in enhancing muscle mass and function even without resistance training in the elderly (97). There is also evidence for an effect of creatine on cognitive function, which may be of benefit in the elderly (98). As an ingredient in formulations, creatine could be a useful adjunct to protein. There have been concerns raised regarding creatine and renal function, but there is no evidence indicating that this may be the case (99, 100). Interestingly, a beneficial effect of creatine has been shown in medium- to high-quality trials in patients with certain myopathies (101). Longer term trials employing creatine supplementation in populations such as the elderly, with adequate monitoring of renal function, would be of interest. Such trials would not have to include higher dose or “loading” phases of creatine but merely a chronic lower dose (3–5 g/day) of creatine to have an impact.

## Adverse Effects of Dietary Protein

Increased dietary protein is thought to have impact on renal function (102). What needs to be recognized is that the thesis that dietary protein is causative for renal disease is not supported by evidence (44). Both the WHO (103) and the US Institute of Medicine (8) in setting the requirements for protein have stated, “…that the protein content of the diet is not responsible for the progressive decline in kidney function with age” (p. 842) (8) and, “…protein restriction lowers glomerular filtration rate, suggesting that the decline of glomerular filtration rate with age is a natural consequence of the decline in protein intake as age progresses, and is unrelated to deterioration of renal function” (p. 224) (103). For persons with chronic kidney disease (CKD), lower protein diets have been recommended as being “nephroprotective” (104, 105). Current guidelines for those with CKD are to prescribe a lower protein diet, varying from as low as 0.3–0.9 g/kg/day, dependent on disease stage (106, 107). Of note is that dietary acid load, which is related to protein intake, may be one of the key factors to consider as opposed to protein load, at least in those with earlier stage (3 or less) (105, 108, 109). It would seem prudent, on a low-protein intake even in patients with CKD, to emphasize the highest quality proteins possible (Table 1), due to the need for IAA. Trials of high-quality proteins, or ketoacid supplements, and potential combination with alkali-promoting foods or supplements (108, 109) would be a good avenue for future research.

Lines of evidence from epidemiological studies have led to the thesis that protein causes reductions in bone health (110). More recent evidence, however, favors the concept that protein is a bone health-supporting nutrient (111–113). In addition, meta-analysis of superior quality calcium balance studies has shown that increasing dietary protein actually increases the absorption of calcium (114). Thus, it appears that rather than a protein-induced acid load causing bone calcium resorption, increased dietary protein results in increased intestinal calcium absorption and has no net effect on bone calcium content or fracture risk (115). Applying Hill’s criteria for epidemiologic causation and in a systematic review and meta-analysis, Fenton et al. (116) concluded, “A causal association between dietary acid load and osteoporotic bone disease is not supported by evidence and there is no evidence that an alkaline diet is protective of bone health.” Kerstetter et al. (111) have emphasized that protein may be only supportive of bone health when other nutrients such as calcium and vitamin D are consumed at recommended intake levels.

## Conclusion

Current protein requirements may not be sufficient for older persons. Importantly, there may be benefits associated with higher than minimal protein intakes, such as preservation of LBM and function, which are worth further investigation. The amino acid leucine appears to be a key in terms of triggering a rise in MPS, and thus its content in proteins would be worth considering when evaluating proteins for their ability to support retention of muscle mass. Older persons appear to require higher intakes of leucine to stimulate MPS than younger persons. It may be that higher per-meal intake of protein (leucine) is also worthy of further study as per-meal protein intakes are associated with improved muscle mass and function at least in observational data. Critical illness is another area where we see clinically relevant outcome data with higher protein intakes, for which there is a good theoretical framework. Consensus recommendations for pressure ulcers are that patients consume 1.2–1.5 g/kg/day (76); however, there appears to be no clear benefit of protein–energy supplementation on mitigating pressure ulcer development or healing although the data are from low-quality trials and none have studied protein supplementation (i.e., over and above requirements or current intakes) *per se*. Currently, trials of acute illness are too few to make a recommendation on a potential benefit of protein. Protein- and energy-containing supplements do have measurable impact in certain chronic conditions such as COPD, but are more likely to have an effect in persons who are malnourished/undernourished and in persons who are sarcopenic. The impact of protein supplementation on functional outcomes is inconclusive at present, and it is likely that functional improvements are far more likely to be seen with supplement trials when combined with exercise and/or in persons with impaired function. Certain ingredients show promise as adjuncts to protein in aiding in augmenting and/or preserving lean mass and function. The impact of β-HMB appears limited in older persons, with the potential exception of recovery from bed rest. Supplemental creatine is a promising ingredient that appears able to augment not only LBM but also function and ADL. There is no *bona fide* evidence linking dietary protein to the actual development of renal disease. Those patients with preexisting renal disease are advised to manage their protein intake and dietary acid load in accordance with current guidelines (107). As opposed to dietary protein promoting bone loss, it is actually a bone-supporting nutrient but likely only when calcium (1–1.2 g/day) and vitamin D (600 IU) intakes are sufficient.

## Author Contributions

SP conceived, wrote, and prepared the entire manuscript and bears final responsibility for all content of the manuscript.

## Conflict of Interest Statement

SP has received honoraria, travel expenses, and research support from the US National Dairy Council.
