# Corrigendum: Current concepts and unresolved questions in dietary protein requirements and supplements in adults

**DOI:** 10.3389/fnut.2022.1078528

**Published:** 2023-04-12

**Authors:** Stuart M. Phillips

**Affiliations:** McMaster University, Hamilton, ON, Canada

**Keywords:** sarcopenia, critical illness, chronic illness, lean body mass, leucine, creatine

In the published article, there was a typographical error in Table 1 as published. The corrected Table 1 and its caption appear below.

**Table 1:** PDCAAS and DIAAS scores for selected isolated proteins and foods.

**Food**	**PDCAAS**	**DIAAS**	**Limiting AA**
MPC^1^	1.00	1.18	Met + Cys
WPI^1^	1.00	1.09	Val
SPI^1^	0.98	0.90	Met + Cys
PPC^1^	0.89	0.82	Met + Cys
RPC^1^	0.42	0.37	Lys
Whole milk^2^	1.00	1.43	Met + Cys
Cooked peas^1^	0.58	0.82	Met + Cys
Cooked rice^1^	0.62	0.59	Lys
Almonds^5^	0.35	0.40	Lys
Chickpeas^6^	0.52	0.67	Trp
Tofu^3, 4^	0.70	0.97	Met + Cys
Corn-based cereal^1^	0.08	0.01	Lys
Hydrolyzed collagen^7^	0.0	0.0	Trp

PDCAAS, protein digestibility-corrected amino acid score; DIAAS, digestible indispensable amino acid score for adolescents and adults; MPC, milk protein concentrate; WPI, whey protein isolate; SPI, soy protein isolate; PPC, pea protein concentrate; RPC, rice protein concentrate.

1 Values from Rutherfurd et al. (1).

2 Values from FAO (2) and Herreman et al. (3).

3 Values for PDCAAS from Anwar and El-Chaghaby (4).

4 Values for DIAAS from Reynaud et al. (5).

5 Values from Ahrens et al. (6).

6 Values from Nosworthy et al. (7).

7 Hydrolyzed collagen has a PDCAAS and DIAAS score of 0 since it contains no tryptophan (Trp) and is very low in methionine (8).

The author apologizes for this error and states that this does not change the scientific conclusions of the article in any way. The original article has been updated.
